# Obituary: Jonas Blomberg (1944–2019)

**DOI:** 10.1186/s12977-019-0469-y

**Published:** 2019-03-14

**Authors:** Patrik Medstrand, Patric Jern

**Affiliations:** 1 Clinical Virology, Department of Translational Medicine, Lund University, Malmö, Sweden; 2 Clinical Microbiology, Skåne University Hospital, Lund, Sweden; 3 Science for Life Laboratory, Department of Medical Biochemistry and Microbiology, Uppsala University, Uppsala, Sweden

In memory of Professor Emeritus Jonas Blomberg, Uppsala, Sweden.

It is with great sadness that we write about the untimely demise of Professor Emeritus Jonas Blomberg, Uppsala, Sweden. Jonas passed away suddenly in the middle of his walk on 5 February 2019 at the age of 75.

**Figure:**
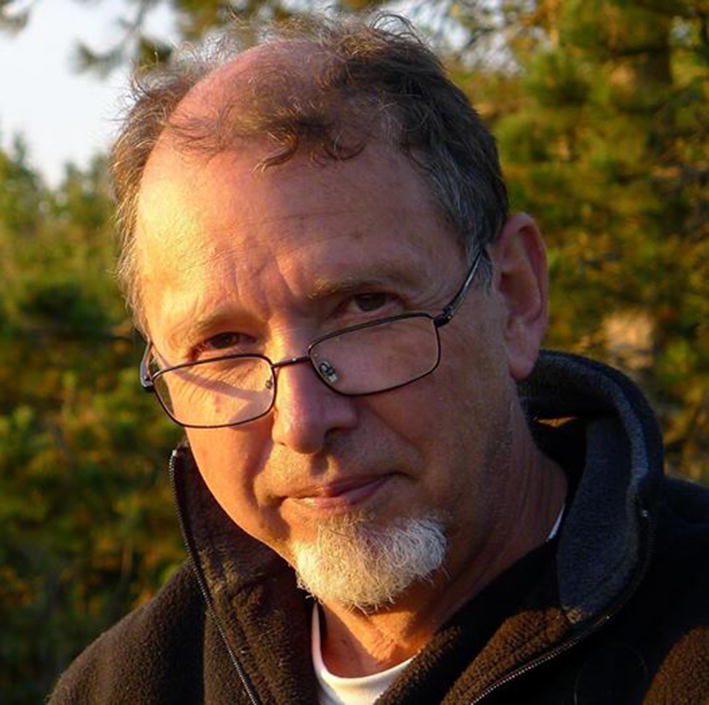
Jonas Blomberg

Throughout his life, Jonas showed a strong commitment to research with training in medicine from Gothenburg University where he attained his MD degree in 1974, followed by a PhD in medical biochemistry in 1977. Jonas then worked as a laboratory physician at the Sahlgrenska Hospital, Gothenburg, Sweden, and was board certified in Clinical Virology in 1979. That same year, he was introduced to retrovirology when he joined Dr. John Stephenson’s laboratory at the National Cancer institute, Frederick, MD, USA, to work on retroviruses as a postdoctoral fellow between 1979 and 1981. After his postdoctoral tenure, he returned to Sweden as an Associate Professor in Virology at Lund University, where he also held a position as Senior Laboratory Physician in Clinical Virology until he moved to Uppsala University in 1996 as full Professor and Chair of Clinical Virology at the Medical Faculty. Since 2011, Jonas continued his research as Professor Emeritus.

Jonas’ research involved several lines of investigation and frequently connected with his clinical work with much focus on diagnosis of microbial disease and development of broadly targeted multiplex techniques for detection of pathogen nucleic acids as well as antibodies to pathogens. Naturally, his work also involved the then newly identified HIV, and later extended to cover HTLV. As well, he had great interest in determining the evolutionary relationships between these and related exogenous retroviruses and endogenous retrovirus (ERV) counterparts in vertebrate host genomes.

During his career, Jonas frequently attended workshops and meetings, including the annual Retrovirus meeting at Cold Spring Harbor, as well as specialized meetings on ERVs. He was an active participant at these meetings, promoting and contributing results and opinions to the scientific community, as well as introducing his doctoral students (including ourselves) and colleagues to the expanding research on retroviruses and ERVs.

While much of Jonas’ research was conducted using molecular tools developed in the laboratory with clinical connections to characterize retroviruses and ERVs [1], bioinformatics methodology gradually assumed a larger role in his research. The increasing availability of host genomic sequences facilitated identification and analyses of ERVs that motivated development of a specialized ERV analysis tool, RetroTector [2], which has proved useful for example to conduct retrovirus and ERV phylogenetic analyses [3] and continues to demonstrate use by the larger research community.

Based on his long experience in retrovirus research, Jonas also contributed his expertise to the retrovirus study group at the International Committee on Taxonomy of Viruses (ICTV) and its proposed updated nomenclature in 2018 [4]. During recent years, Jonas shifted much of his attention towards encephalomyelitis/chronic fatigue syndrome (ME/CFS) [5], and he was an appreciated spokesman for improved diagnosis and development of appropriate treatment of these patients.

Jonas never truly retired, remaining committed to the study of infectious diseases and retroviruses. He will be greatly missed by the retrovirology community.
